# Correlation of procalcitonin and c-reactive protein levels with pathogen distribution and infection localization in urinary tract infections

**DOI:** 10.1038/s41598-023-44451-6

**Published:** 2023-10-11

**Authors:** Jing Shi, Zhi-Song Zhan, Zu-Shun Zheng, Xue-Xia Zhu, Xin-Yi Zhou, Shi-Yan Zhang

**Affiliations:** 1 Fuding Hospital, Fujian University of Traditional Chinese Medicine, Fuding, 355200 Fujian China; 2 Department of Clinical Laboratory, Fuding Hospital, Fujian University of Traditional Chinese Medicine, 120 South Road of Old City, Fuding, China

**Keywords:** Biomarkers, Urology

## Abstract

Aimed to explore the relationships between infection localization, bacterial species, and procalcitonin (PCT) and C-reactive protein (CRP) levels in urinary tract infections (UTIs). A retrospective study included 314 UTI hospitalized patients divided into two groups (268 with lower UTI, 46 with upper UTI) in a tertiary care hospital. PCT and CRP were performed. Bacterial isolates were identified using standard microbiological techniques, and statistical analyses were performed to assess associations between infection localization, bacterial species, PCT, and CRP levels. Age and gender showed no significant differences between the lower and upper UTIs. *Escherichia coli* dominated as the leading UTI pathogen. A positive correlation (r = 0.646,* P* < 0.001) between PCT and CRP levels was found. The subgroup with ureteritis in the upper UTI category exhibited the highest PCT and CRP levels. PCT and CRP exhibited favorable diagnostic potential in predicting upper UTIs, with AUCs of 0.644 and 0.629, respectively. The optimal cutoff values were 0.21 ng/mL for PCT and 60.77 mg/L for CRP. Sensitivities were 69.03% and 77.99%, while specificities were 56.52% and 47.83%, respectively. *E. coli* emerged as the predominant bacterium in UTIs. PCT and CRP demonstrated moderate diagnostic efficacy in distinguishing between upper and lower UTIs. Notably, PCT and CRP exhibited enhanced utility in identifying ureteritis.

## Introduction

Urinary tract infections (UTIs) are among the most prevalent bacterial infections worldwide, affecting people of all ages and genders^1^. UTIs can vary in severity and location, ranging from lower urinary tract infections (cystitis and urethritis) to more severe upper urinary tract infections (pyelonephritis and ureteritis)^2^. The clinical presentation of UTIs can be diverse, posing challenges in both accurate diagnosis and effective management. Complicating matters further, UTIs are caused by a spectrum of bacterial species, each potentially requiring distinct treatment strategies^3^.

To address these challenges and enhance our understanding of UTIs, this study delved into the intricate relationships between infection localization, bacterial species, and the levels of two biomarkers: procalcitonin (PCT) and C-reactive protein (CRP). The significance of identifying trends in infection characteristics and levels of PCT and CRP cannot be overstated. Effective clinical decision-making relies on accurate diagnosis, especially in cases where UTIs are complicated by factors such as antimicrobial resistance^4^. Moreover, as UTIs can present with varying clinical symptoms, understanding the connection between PCT and CRP levels and infection sites holds potential for improved patient stratification and personalized treatment approaches. PCT and CRP have garnered attention as potential indicators of infection severity and response to treatment^5^. PCT and CRP are inflammatory biomarkers that have been shown to support diagnosis and monitoring of (bacterial) respiratory tract infections and sepsis^6,7^. While limited studies suggest their usefulness in supporting the sites of UTI diagnosis, their performance in different UTI scenarios remains a subject of investigation.

This study employed a retrospective observational design, drawing upon electronic medical records of UTI patients within a tertiary care hospital. The dataset encompassed a diverse range of UTI presentations, spanning from lower to upper urinary tract infections. The integration of demographic data, bacterial culture results, and biomarker measurements aimed to unravel correlations, associations, and trends within this intricate landscape.

## Results

A total of 314 consecutive hospitalized patients were included in this study, including 122 males (38.9%) and 192 females (61.1%), 268 lower UTI group and 46 upper UTI group. The distribution of age and gender across the lower and upper UTI groups was analyzed using the Mann–Whitney *U*-test and Chi-square test, respectively. The results indicated that there were no statistically significant differences in age between the two groups (*P* = 0.128). Similarly, the gender distribution was comparable between the lower and upper UTI groups (*P* = 0.712) (Table 1).

**Table 1:** Comparison of age and gender distribution between lower and upper UTIs.

Items	Lower UTI (n = 268)	Upper UTI (n = 46)	Mann–Whitney *U*-test/*χ*^2^	*p-*value
Age median (IQR) (year)	69.5 (58.0–76.0)	67.5 (51.0–77.0)	− 1.521	0.128
Age range	1.0–95.0	23.0–86.0		
Gender, n (%)			0.136	0.712
Male (122)	103 (38.4)	19 (41.3)		
Female (192)	165 (61.6)	27 (58.7)		

Shapiro–Wilk test was used to assess the age distribution within the lower and upper UTIs groups, resulting in the following outcomes: PCT (positive: *Z* = 0.345, *P* < 0.001; negative: *Z* = 0.342, *P* < 0.001); CRP (positive: *Z* = 0.215, *P* < 0.001; negative: *Z* = 0.163,* P* < 0.001). These results indicated a non-normal distribution of age data.

*UTIs* urinary tract infections.

**Figure 1:**
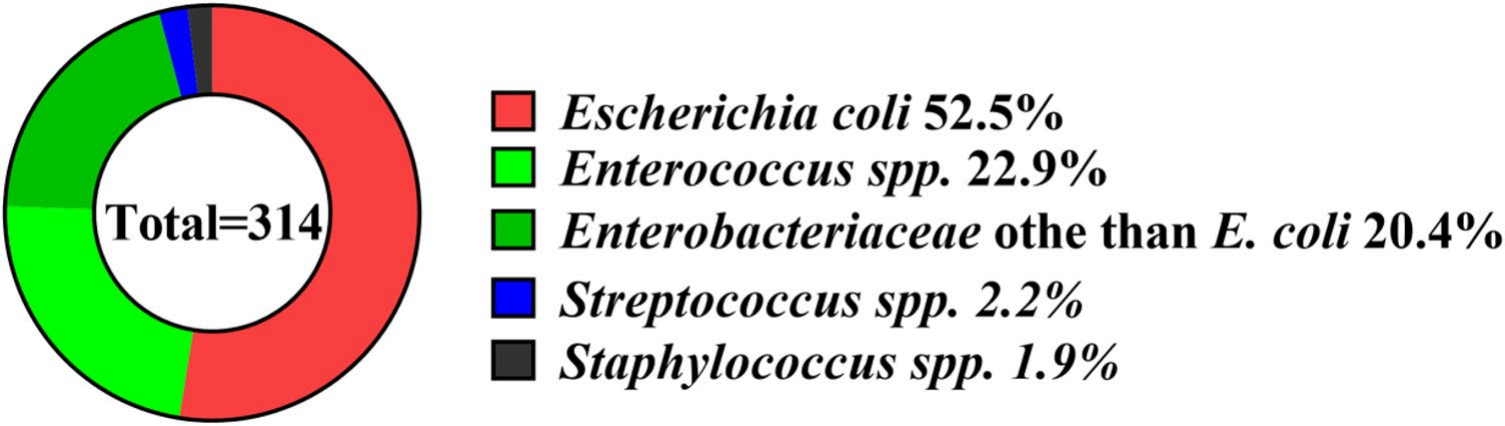
The distribution of the UTI pathogen species.

In the urine samples of 314 hospitalized patients, a total of 314 strains of bacteria were isolated, including 85 Gram-positive strains (27.1%) and 229 Gram-negative strains (72.9%). The pathogenic bacteria corresponding to their phylogenetic relationships were *E. coli* (165 strains), *Enterococcus spp.* (72 strains), *Enterobacteriaceae* other than *E. coli* (64 strains), *Streptococcus spp*. (7 strains), and *Staphylococcus spp*. (6 strains), accounting for 52.5%, 22.9%, 20.4%, 2.2%, and 1.9%, respectively (see Fig. 1).

To assess the relationship between PCT and CRP levels, we calculated the correlation coefficient between the two markers using the spearman test. The correlation coefficient r = 0.646 (*P* < 0.001). This showed a significant positive correlation between PCT and CRP levels.

The examination of PCT and CRP levels across different infection sites revealed intriguing patterns. A Kruskal–Wallis H test was performed to compare the levels of PCT and CRP among various infection site subgroups. The results showed that there were statistically significant differences in PCT and CRP levels among the different infection sites (*P* = 0.002 for PCT, *P* = 0.005 for CRP). Notably, further analysis demonstrated significant differences in PCT and CRP levels between the Urethritis and Ureteritis subgroups (PCT: *Z* = – 3.352, *P* = 0.005; CRP: *Z* = – 3.448, *P* = 0.003) (Table 2).

**Table 2:** Procalcitonin and CRP levels associated with various infection sites.

Site	Number (n = 314) (%)	PCT (IQR) (ng/mL)	CRP (IQR) (mg/L)
Urethritis	258 (82.2)	0.12 (0.08–0.37)	14.98 (4.90–52.35)
Cystitis	10 (3.2)	0.12 (0.09–6.38)	18.14 (4.90–134.79)
Pyelonephritis	36 (10.8)	0.17 (0.09–1.13)	24.02 (5.48–103.92)
Ureteritis	12 (3.8)	3.15 (0.37–31.27)	92.32 (49.19–124.93)
H-value		14.701	12.660
P-value		0.002	0.005

A Kruskal–Wallis H test was performing between the four subgroups. A statistically significant difference was observed only in the subgroup of ureteritis. Further analysis exhibited significant differences between the urethritis and ureteritis subgroups for both CRP (*Z* =  − 3.352, *P* = 0.005) and PCT (*Z* =  − 3.448, *P* = 0.003).

*IQR* interquartile range, *PCT* procalcitonin, *CRP* C-reactive protein.

The comparison of PCT and CRP levels between the lower and upper UTI groups using the Mann–Whitney *U*-test indicated statistically significant differences. The median PCT levels were 0.12 (IQR 0.08–0.37) for the lower UTI group and 0.26 (IQR 0.09–6.21) for the upper UTI group (*P* = 0.002). Similarly, the median CRP levels were 14.98 (IQR 4.90–52.48) for the lower UTI group and 50.12 (IQR 6.82–118.72) for the upper UTI group (*P* = 0.005) (Table 3). These significant differences in PCT and CRP levels between lower and upper UTI groups underscored their potential as diagnostic tools, suggesting their role in distinguishing between different UTI presentations.

**Table 3:** Comparison of PCT and CRP levels between lower and upper UTIs.

	PCT (IQR) (ng/mL)	CRP (IQR) (mg/L)
Lower UTI (n = 268)	0.12 (0.08–0.37)	14.98 (4.90–52.48)
Upper UTI (n = 46)	0.26(0.09–6.21)	50.12 (6.82–118.72)
Z-value	− 3.129	− 2.811
P-value	0.002	0.005

Mann–Whitney *U*-test was carried out to compare the results between the lower and upper UTIs groups.

*IQR* interquartile range, *PCT* procalcitonin, *CRP* C-reactive protein, *UTIs* urinary tract infections.

**Figure 2:**
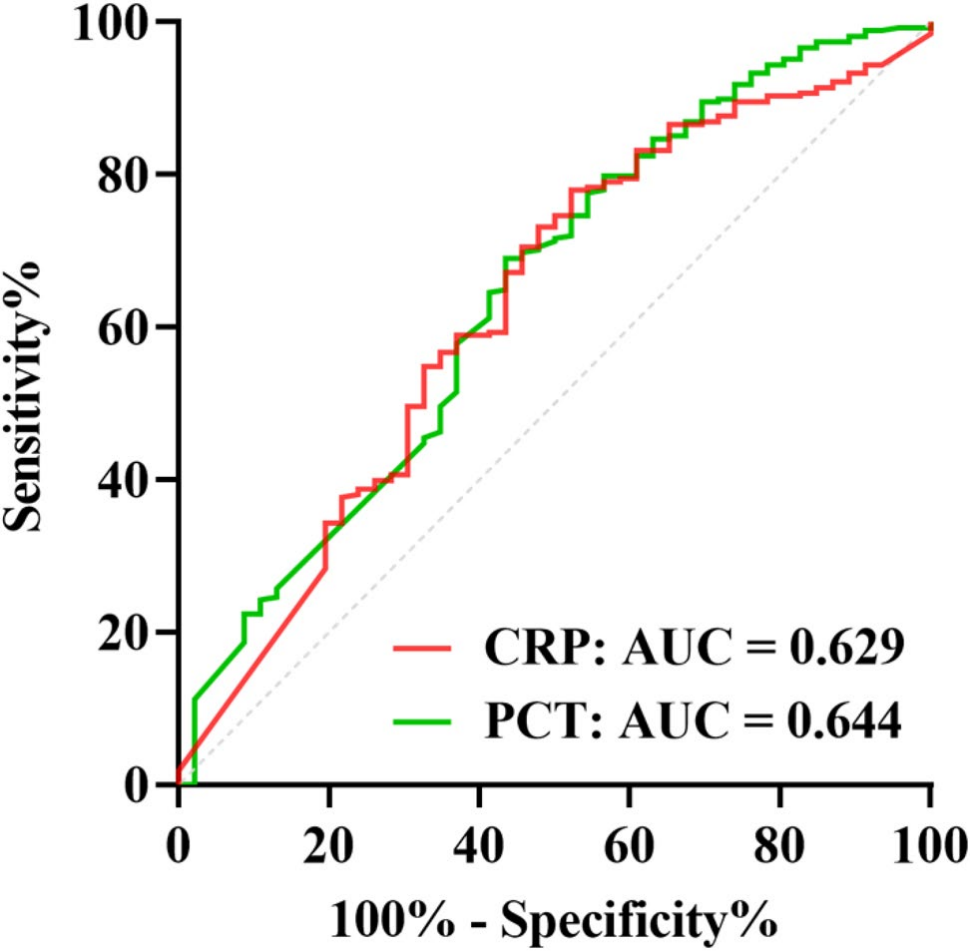
ROC curve comparing PCT and CRP levels in upper UTI versus lower UTI groups.

The diagnostic performance of PCT and CRP in predicting upper UTI bacteria was evaluated using receiver operating characteristic (ROC) curves. The area under the curve (AUC) for PCT was 0.644 (95% CI 0.540–0.734), and for CRP, it was 0.629 (95% CI 0.538–0.719). The optimal cutoff values were determined, and sensitivity, specificity, positive predictive value, negative predictive value, positive likelihood ratio, negative likelihood ratio, and accuracy were calculated. The statistical analysis showed that both PCT and CRP exhibited significant predictive potential for upper UTI bacteria (PCT: *P* = 0.002, CRP: *P* = 0.005) (Table 4 and Fig. 2).

**Table 4:** Test performance of PCT and CRP for prediction of upper UTI bacteria.

Item	PCT	CRP
Optimal cutoff	0.21 ng/mL	60.77 mg/L
AUC (95% CI)	0.644 (0.540–0.734)	0.629 (0.538–0.719)
Standard error	0.046	0.046
Sensitivity, % (95% CI)	69.03 (63.26–74.26)	77.99 (72.65–82.53)
Specificity, % (95% CI)	56.52 (42.25–69.79)	47.83 (34.12–61.86)
Accuracy, % (95% CI)	58.3 5(45.33–70.44)	52.25 (39.76–64.89)
Diagnostic odds ratio, % (95% CI)	2.90 (1.26–6.66)	3.25 (1.3 8- 7.66)
Positive predictive value, % (95% CI)	21.41 (15.83–29.67)	20.42 (15.92–27.08)
Negative predictive value, % (95% CI)	92.85 (90.94–94.38)	95.38 (93.93–96.49)
Positive likelihood ratio, (95% CI)	1.85 (1.1 0–2.46)	1.49 (1.10–2.16)
Negative likelihood ratio, (95% CI)	0.55 (0.37–0.87)	0.46(0.28–0.80)
Youden’s index (%)	25.56	25.82
*P*-value	0.002	0.005

Optimal cutoff values derived from receiver operating characteristics by Youden’s index.

*AUC* area under the receiver operating characteristic curve, *95*% *CI* 95% confidence interval, *PCT* procalcitonin, *CRP* C-reactive protein, *UTI* urinary tract infection.

The Mann–Whitney *U*-test was employed to compare PCT and CRP levels between Gram-positive and Gram-negative bacteria groups. The analysis showed no statistically significant differences between the two groups (PCT: *P* = 0.726, CRP: *P* = 0.639). Moreover, an in-depth analysis of PCT and CRP levels among diverse pathogen species was executed through the utilization of the Kruskal–Wallis H test. However, the results indicated no significant differences in these levels across various species (Tables 5 and 6).

**Table 5:** Pathogen-specific comparison of PCT and CRP levels.

Pathogen	PCT (IQR) (ng/mL)	CRP (IQR) (mg/L)
Gram-positive bacteria (85)	0.13 (0.09–0.47)	17.68 (4.90–68.02)
Gram-negative bacteria (229)	0.12 (0.09–0.47)	17.18 (4.98–58.83)
Z-value	− 0.351	− 0.469
P-value	0.726	0.639

Mann–Whitney *U*-test was carried out to compare the PCT and CRP levels between gram-positive and gram-negative bacteria groups.

*IQR* interquartile range, *PCT* procalcitonin, *CRP* C-reactive protein.

**Table 6:** Pathogen species in the distribution of lower and upper UTIs [case (%)].

Pathogen species	Lower UTIs (268)	Upper UTIs (46)	*χ*^2^	*P*-value
Gram-positive bacteria	70 (26.1)	15 (32.6)	0.837	0.372
*Escherichia coli* (165)	144 (53.7)	21 (45.7)	1.028	0.340
*Enterococcus spp.* (72)	61 (22.8)	11 (23.9)	0.029	1.000
*Enterobacteriaceae* other than *E. coli* (64)	54 (20.1)	10 (21.7)	0.061	0.843
*Streptococcus spp.* (7)	4 (1.5)	3 (6.5)	–	0.067*
*Staphylococcus spp.* (6)	5 (1.9)	1 (2.2)	–	1.000*

*UTIs* urinary tract infections.

*Fisher’s exact test.

A Kruskal–Wallis H test was conducted to compare the levels of PCT and CRP among various pathogen species subgroups. The results revealed that there were no statistically significant differences in PCT and CRP levels among the various pathogen species (*P* = 0.634 for PCT, *P* = 0.230 for CRP) (Table 7).

**Table 7:** PCT and CRP levels associated with different pathogen species.

Pathogen species	Number (314)	PCT (ng/mL)	CRP (mg/L)
*Escherichia coli*	165	0.12 (0.76–0.40)	17.12 (5.06–55.20)
*Enterococcus spp.*	72	0.15 (0.09–0.48)	20.53 (4.90–68.72)
*Enterobacteriaceae* other than *E. coli*	64	0.11 (0.09–0.87)	23.74 (4.90–84.24)
*Streptococcus spp.*	7	0.09 (0.03–0.15)	4.90 (4.90–6.64)
*Staphylococcus spp.*	6	0.12 (0.08–22.41)	40.15 (4.90–113.15)
*H*-value	–	2.560	5.618
*P*-value	–	0.634	0.230

The analysis involved *H*–value and *P*–value for each pathogen species, obtained through Kruskal–Wallis *H* test.

*IQR* interquartile range, *PCT* procalcitonin, *CRP* C-reactive protein.

## Discussion

The findings of the study shed light on several key aspects in relation to UTIs, encompassing patient demographics, infection characteristics, bacterial species, PCT and CRP levels. Notably, the retrospective observational design allowed for the analysis of a diverse patient population, offering a realistic depiction of the clinical landscape. The results of this study showed that the top pathogenic bacteria in the urine sample specimens of 314 hospitalized patients was *E. coli* (*165 strains*), accounting for 52.5%, followed by *Enterococcus spp.* (72 strains, 22.9%), *Enterobacteriaceae* other than *E. coli* (64 strains, 20.4%), *Streptococcus spp*. (7 strains, 2.2%), and *Staphylococcus spp*. (6 strains, 1.9%). This is consistent with similar studies^8,9^, which showed that the majority of common bacteria identified in UTIs were Gram-negative bacteria, mainly including *E. coli*, and *E.coli* accounted for the largest proportion of microorganisms identified in the urine samples^10,11^.

The comparison of age and gender distribution between lower and upper UTI groups indicated no statistically significant differences. This suggested that age and gender might not play a prominent role in distinguishing between these two types of UTIs, aligning with previous research that emphasized other factors in infection localization^12^. While age and gender did not exhibit significant differences between lower and upper UTI groups, this finding did not diminish their importance in the realm of UTIs. The lack of substantial age-related variations aligned with some prior studies^13^ that emphasized other contributing factors in infection localization, such as anatomical differences, underlying health conditions, and microbial virulence factors. Similarly, gender-associated disparities in UTI incidence have been widely reported, with this study reaffirming the higher prevalence of UTIs in women. The prevalence of UTI in our study was noted to be higher in women (61.1%). This was consistent with the results of earlier studies conducted by Narayan et al.^14^ and by Narayan et al.^15^, where the prevalence were 60.49% and 66.15%, respectively. The reason for this higher prevalence is because the female urethra is shorter and therefore the perineal and fecal flora can be transmitted over a shorter distance^16^. Therefore, urinary factors, urinary osmolality factors, sexual factors, intraoral factors, vaginal pH and secretory status are responsible for the higher prevalence of UTIs in women^17,18^. These observations underscored the need for multifaceted approaches in UTI management, focusing not solely on age or gender but embracing a holistic perspective.

Investigating the association between infection sites and PCT and CRP offers meaningful perspectives on their potential utility as biomarkers for assessing the severity of UTIs. In alignment with our findings, a prior study conducted by Levine et al.^19^ demonstrated an AUC of 0.717 (95% CI 0.643–0.791, p < 0.001) for PCT in predicting UTIs, along with a sensitivity of 67% and specificity of 63%. Another investigation by Choi et al.^20^ revealed a different AUC for PCT in UTI diagnosis (0.56, 95% CI 0.46–0.65); however, it's noteworthy that this study focused on older adults (≥ 65 years old). A preceding investigation by Xu et al.^21^ highlighted that PCT values exhibited a correlation with the extent of renal involvement, in contrast to CRP values, which did not demonstrate a significant correlation in this context. PCT displayed a sensitivity of 90.47% and specificity of 88% in predicting nephropathia, while CRP exhibited a sensitivity of 85.71% and specificity of 48%. The difference in CRP levels of this study between upper and lower UTIs was significant (*P* = 0.005), and comparable observations have been reported in studies conducted by Narayan et al.^14^ and by Sproston et al.^22^. Both PCT and CRP showed potential in distinguishing between upper and lower urinary tract infections. These consistency with our study results reinforced the valuable role of PCT and CRP as indicators for UTI severity.

The examination of PCT and CRP levels across different infection sites provided intriguing insights. The statistically significant differences in PCT and CRP levels among different infection sites, particularly within the subgroup of ureteritis, underscored the potential of these biomarkers in indicating the severity of infection. Moreover, the ROC analysis demonstrated the diagnostic potential of PCT and CRP levels in predicting upper UTI bacteria, further emphasizing their clinical significance.

Comparison of PCT and CRP levels between Gram-positive and Gram-negative bacteria groups revealed no significant differences. However, an in-depth analysis of various pathogen species indicated distinct biomarker profiles. The variations observed in PCT and CRP levels among different species highlighted the potential of these markers in aiding the identification of specific pathogens, which could have implications for targeted treatment approaches.

### Limitations and future directions

While this study provided valuable insights, several limitations warrant consideration. The retrospective design might introduce bias, and the data were drawn from a single tertiary care hospital, limiting generalizability. Additionally, factors such as comorbidities and medication use were not fully explored. Future research could involve larger and more diverse patient cohorts, along with prospective designs to validate the observed associations.

## Methods

### Study setting and design

This study employed a retrospective observational approach to analyze data extracted from electronic health records of a total of 314 consecutive patients diagnosed with UTIs at a tertiary care hospital (Fuding Hospital, Fujian University of Traditional Chinese Medicine). The patients included 46 upper UTI patients and 268 lower UTI patients. The data collection period spanned from January 2023 to June 2023, encompassing a diverse patient population with varying UTI manifestations. The utilization of a retrospective design allowed for the examination of a wide range of cases, capturing real-world clinical scenarios. This study was conducted according to ethical guidelines and received approval from the Medical Ethics Committee of Fuding Hospital, Fujian University of Traditional Chinese Medicine (Ethics approval number: Fuding Hospital 2023008). Informed consent was obtained by opt-out method. The utmost confidentiality and privacy of patients were rigorously upheld throughout the entire data collection and analysis process.

### Inclusion and exclusion criteria

Inclusion criteria: Patients with three urine samples showing > 10^5^ organisms/mL were included. Clean catch midstream urine samples with appropriate instructions were considered. Exclusion criteria: Patients with inflammatory conditions other than UTIs, tumor, pregnancy.

### Data collection and outcome measures

Patient information, including demographic details such as gender, age, and medical department, was extracted from the electronic medical records. In addition, the levels of PCT and CRP were recorded.

Upon diagnosis of UTI, blood samples were collected to measure PCT and CRP levels. PCT levels were assessed using a chemiluminescent immunoassay (the Cobas e411/E601, Roche Diagnostics, Mannheim, Germany). CRP levels were determined through a high sensitivity turbidimetric immunoassay (The Dimension Vista 1500 Intelligent Lab system, Siemens Healthcare GmbH, Erlangen, Germany). The analyses of PCT and CRP were based on the manufacturer's instructions.

Urine samples were collected by either bladder catheterization or a midstream clean catch method. Pyuria was defined as the presence of more than 5 white blood cells (WBC) per high power field (HPF) in the urine sediment, while significant bacteriuria was identified as the growth of a single pathogenic microorganism exceeding 100,000 colony-forming units (CFU) per milliliter (mL) of urine. Subsequently, bacterial isolates extracted from urine cultures underwent identification processes that incorporated conventional microbiological techniques alongside the utilization of API systems (bioMérieux, BacT/ALERT3D, France). This encompassing approach included methodologies such as Gram staining and biochemical tests. The categorization of bacterial species into either Gram-positive or Gram-negative groups was determined based on their distinctive responses to Gram staining.

Patients were categorized based on infection localization, discerning between lower UTI and upper UTI groups. Notably, only isolates classified as genuine pathogens were included in the analysis. These pathogens were further divided into two major groups: Gram-positive bacteria and Gram-negative bacteria. Within these groups, the pathogens were stratified into five distinct categories corresponding to their phylogenetic relationships: *Escherichia coli*, *Enterococcus spp*., *Enterobacteriaceae* other than *E. coli*, *Streptococcus spp*., *Staphylococcus spp.*

### Statistical analysis

Statistical analysis was carried out using SPSS version 22.0 software (SPSS Inc.). To assess data distribution, the Shapiro–Wilk test was initially employed. Continuous data were presented as medians with interquartile ranges (IQR), while categorical data were expressed as counts and percentages. Given the non-normal distribution observed, subsequent analysis involved nonparametric tests. Intergroup comparisons were performed using the Chi-square test (χ^2^) or Fisher’s exact test. The Mann–Whitney U-test was used to compare age and gender distributions between groups. Spearman’s rank correlation evaluated variable correlations.

Associations between biomarkers and infection sites were assessed using the Kruskal–Wallis H test across subgroups. Significant differences were probed further through post hoc analysis, involving the calculation of *P*–values and interquartile ranges (IQRs) for PCT and CRP levels. Receiver operating characteristic (ROC) curves were constructed, and optimal cutoff values were derived using Youden’s index. Metrics such as sensitivity, specificity, positive predictive value, negative predictive value, positive likelihood ratio, negative likelihood ratio, and accuracy were computed from 2 × 2 tables to gauge performance. Diagnostic odds ratio (DOR) was also used as a measure of effectiveness, which is the ratio of the odds of a positive test in patients with the disease to the odds of a positive test in patients without the disease. Comparisons of PCT and CRP levels between Gram-positive and Gram-negative bacteria, as well as different pathogen species, were executed utilizing the Mann–Whitney *U* test. All statistical tests were conducted at a significance level of *P* < 0.05.

## Conclusions

Through a meticulous exploration of patient demographics, infection characteristics, bacterial species, and biomarker levels, this study contributes to the understanding of UTIs. The insights gained have the potential to inform clinical decision-making, enhance diagnostic accuracy, and guide tailored treatment strategies. Moving forward, continued research in this domain is essential to refine our understanding and optimize patient care.

## Data Availability

The datasets used and/or analysed during the current study available from the corresponding author on reasonable request.
